# Dietary Salt Reduction, Prevalence of Hypertension and Avoidable Burden of Stroke in Vietnam: Modelling the Health and Economic Impacts

**DOI:** 10.3389/fpubh.2021.682975

**Published:** 2021-06-04

**Authors:** Leopold Ndemnge Aminde, Hai N. Phung, Dung Phung, Linda J. Cobiac, J. Lennert Veerman

**Affiliations:** ^1^School of Medicine, Griffith University, Gold Coast, QLD, Australia; ^2^Nuffield Department of Population Health, University of Oxford, Oxford, United Kingdom

**Keywords:** sodium, blood pressure, stroke, mortality, multi-state model, healthcare costs

## Abstract

Dietary salt reduction has been recommended as a cost-effective population-wide strategy to prevent cardiovascular disease. The health and economic impact of salt consumption on the future burden of stroke in Vietnam is not known.

**Objective:** To estimate the avoidable incidence of and deaths from stroke, as well as the healthy life years and healthcare costs that could be gained from reducing salt consumption in Vietnam.

**Methods:** This was a macrosimulation health and economic impact assessment study. Data on blood pressure, salt consumption and stroke epidemiology were obtained from the Vietnam 2015 STEPS survey and the Global Burden of Disease study. A proportional multi-cohort multistate lifetable Markov model was used to estimate the impact of achieving the Vietnam national salt targets of 8 g/day by 2025 and 7 g/day by 2030, and to the 5 g/day WHO recommendation by 2030. Probabilistic sensitivity analysis was conducted to quantify the uncertainty in our projections.

**Results:** If the 8 g/day, 7 g/day, and 5 g/day targets were achieved, the prevalence of hypertension could reduce by 1.2% (95% uncertainty interval [UI]: 0.5 to 2.3), 2.0% (95% UI: 0.8 to 3.6), and 3.5% (95% UI: 1.5 to 6.3), respectively. This would translate, respectively, to over 80,000, 180,000, and 257,000 incident strokes and over 18,000, 55,000, and 73,000 stroke deaths averted. By 2025, over 56,554 stroke-related health-adjusted life years (HALYs) could be gained while saving over US$ 42.6 million in stroke healthcare costs. By 2030, about 206,030 HALYs (for 7 g/day target) and 262,170 HALYs (for 5 g/day target) could be gained while saving over US$ 88.1 million and US$ 122.3 million in stroke healthcare costs respectively.

**Conclusion:** Achieving the national salt reduction targets could result in substantial population health and economic benefits. Estimated gains were larger if the WHO salt targets were attained and if changes can be sustained over the longer term. Future work should consider the equity impacts of specific salt reduction programs.

## Introduction

Hypertension is the single largest risk factor for cardiovascular disease (CVD), the leading cause of death of globally (1). A global synthesis of national population-based surveys found that the number of people with hypertension has more than doubled over the last four decades, with an estimated 1.1 billion people with the condition in 2015 (2). The majority of affected people are in the low-income and middle-income countries (LMIC), where increasing trends in age-standardised mean systolic blood pressures (SBP) have been found (2). In Vietnam, a recent systematic review of population-based studies of measured hypertension revealed that over one in five (21.1%) adults had hypertension (3). However, using a broader definition including measured SBP ≥ 140/90 mmHg and/or self-report physician diagnosed and/or currently on blood pressure lowering medication, the national WHO STEPS survey reported a prevalence of 30.6% in 30–69-year-old adults (4). In general, hypertension is more common in men, increases with age, with prevalence over 40% in those aged 40 years and more (4, 5). A significant proportion of people with hypertension in Vietnam are undiagnosed, with studies reporting low awareness, treatment, and control rates (5, 6).

Hypertension is described as a silent killer because it generally has no symptoms. By the time an individual is symptomatic, they most likely have developed one or more complications. These may include myocardial infarction, stroke, hypertensive heart disease, heart failure, chronic kidney disease and retinopathy (1). In Vietnam, stroke is the most frequent of these hypertension-related complications. According to a hospital-based retrospective analysis in central Vietnam, the age-standardised incidence rate of first ever stroke was 115.7 per 100,000 persons between 2009 and 2011 (7). Stroke is also the leading cause of death in the country. In 2019, over one in five (21.5%) deaths, that is, 136,000 deaths in Vietnam were due to stroke, representing an almost 2% increase from two decades earlier (8). In addition, stroke was responsible for over three million disability adjusted life years in 2019 in Vietnam, representing a 13% increase compared to a decade earlier (8). The high rates of stroke incidence and mortality in Vietnam have mostly been attributed to undiagnosed and poorly treated or uncontrolled hypertension (7).

Among the myriad of modifiable risk factors known to increase an individual's risk of developing hypertension, excessive sodium (salt) consumption is a major contributor. By raising an individual's blood pressure, it increases the risk of developing cardiovascular events (9). Mozaffarian et al. showed that sodium intake above 2 grammes/day was responsible for over 1.6 million CVD deaths worldwide in 2010, majority from stroke and ischaemic heart disease (10). Salt consumption globally is well above recommended levels. Asian countries have some of the highest levels of salt consumption. In Vietnam, a national survey using spot urine samples estimated that the average salt intakes in adults was around 9.4 grammes per day (10.5 g/day in men and 8.3 g/day in women) (4). This is nearly twice the current World Health Organisation recommended consumption of <5 g of salt per day (11).

There is strong evidence that reducing average salt intake decreases blood pressure which could prevent thousands of future cardiovascular events across populations (9, 12). A recent comprehensive review on the economic evaluation of strategies to prevent CVD in LMICs revealed that population-wide salt reduction was cost-saving. However, the authors underscored the need for more country-specific studies, given the dearth in such studies especially from Africa and Asia (13). Studies from high-income countries have shown that salt reduction at population levels saves thousands of lives and has good value for money (14, 15). However, very few of such studies have been conducted in low-income and middle-income countries (16). Given the very high levels of salt intake, as well as high rates of stroke events in Vietnam, it is likely that such strategies could yield significant health and economic benefits.

In 2018, the Prime Minister of Vietnam approved “The Healthy Vietnam Program” whose general objective is to “…improve the health, stature, longevity, and quality of life of the Vietnamese people” (17). Among the targets outlined in the first objective of this program, is to reduce salt consumption per person per day to <8 grammes by 2025 and <7 grammes by 2030. This study therefore seeks to quantify: (i) the new cases of and deaths from (absolute and relative) stroke that would be prevented; (ii) the number of health adjusted life years that would be gained; and (iii) the future healthcare costs from stroke cases that could be avoided, if these salt reduction targets were achieved.

## Materials and Methods

### Study Population and Data Sources

This study included the entire cohort of Vietnamese adults aged ≥ 25 years in 2019 (base year), which included 61 million people (29.5 million men and 31.5 million women). Persons below 25 years were excluded given the comparatively low rates of blood pressure-related stroke events, and the absence of reliable data on the risk-outcome relation between blood pressure and stroke in this sub-population.

Data on blood pressure and salt intake was obtained from the 2015 National Survey on Risk factors of Non-communicable Diseases for Vietnam (4). This nationally representative survey used a complex multi-stage sampling process to include adults aged ≥18 years from across all 63 provinces/cities of Vietnam. Standard blood pressure measurement procedures were used, and 24 h urinary sodium was estimated using the INTERSALT formula from the spot urine samples collected. This survey applied the methods and tools of the World Health Organisation (WHO) STEPwise approach to NCD surveillance (STEPS) and further details can be found here (4). Data on relative risks as well as baseline epidemiological data (incidence, prevalence, mortality) for stroke in Vietnam were obtained from the Global Burden of Disease (GBD) 2019 study (18). For healthcare costs, data was from a cohort study (19) conducted at the reference Stroke Unit of the 115 People's Hospital. This is a major teaching hospital in Ho Chi Minh city and the study recruited first ever stroke patients with complete electronic records on direct medical costs. A bottom-up costing approach was used in this study and captured the following cost components: consultant fees, bed-day fees, diagnostic imaging, laboratory tests, medication, rehabilitation, minor procedures, medical consumables, and special meals/feeding.

### Modelling Framework

The multiple cohort proportional multistate lifetable (PMSLT) Markov model was used for this analysis (20). It contains three major linked parts: the risk factor (exposure) section, the main lifetable, and the disease Markov sub-models. This model framework is unique given its ability to deal with comorbidity and has been used previously in similar NCD preventive modelling (21, 22). A three-staged process is used: first, modelling the impact of sodium (salt) on blood pressure; second, the impact of changes in blood pressure on stroke incidence and mortality; and third, the impact of changes in mortality on (healthy) life years.

#### Risk Factor Modelling

We used evidence from a meta-analysis of long-term trials (23) on the relationship between salt intake and blood pressure to model the impact of changes (reduction) in population salt intake on mean blood pressure. In this study, a 4.4 grammes reduction in salt intake led to a 4.18 mmHg reduction in systolic blood pressure. This meta-analysis was used given that it included trials with a minimum duration of 1 month, which is the recommended duration when assessing the public health impact of salt reduction (9, 12). We accounted for the differential impact of salt reduction for people with hypertension and those without. Assuming a normal distribution, we estimated the changes in prevalence of hypertension from shifting the blood pressure distribution with and without salt reduction. The “distributions shift” method of the potential impact fraction (PIF)—was used to calculate the proportional change in the incidence of stroke due to changes in the risk factor (blood pressure) distribution. For this computation, we used relative risks from the GBD linking SBP and both forms of stroke, as shown in the Box 1 below. The shift in the population's SBP distribution leads to the re- estimation of stroke incidence, which feeds into the disease Markov models described below.

Box 1 **Distribution shift potential impact fraction formula.**

$$PIF=\frac{\int_{a}^{b}RR(x)P(x)dx-(\int_{a1}^{b1}RR(x)P*(x)dx+\int_{a2}^{b2}RR(x)P*(x)dx)}{\int_{a}^{b}RR(x)P(x)dx}$$

*Where, x* = *SBP exposure levels, RR(x)* = *relative risk, P(x)* = original SBP distribution, P* = *SBP distribution after the intervention, a* = *start integration limit (90 mmHg), b* = *end integration limit (220 mmHg), a1* = *start integration limit for normotensive people (90 mmHg), b1* = *end integration limit for normotensive people (139.9 mmHg), a2* = *start integration limit for hypertensive people (140 mmHg), b2* = *end integration limit for hypertensive people (220 mmHg). A theoretical minimum SBP of 115 mmHg, which is the lowest level considered for elevated CVD risk*.

#### Disease Markov and Multistate Lifetable Modelling

**Figure 1:**
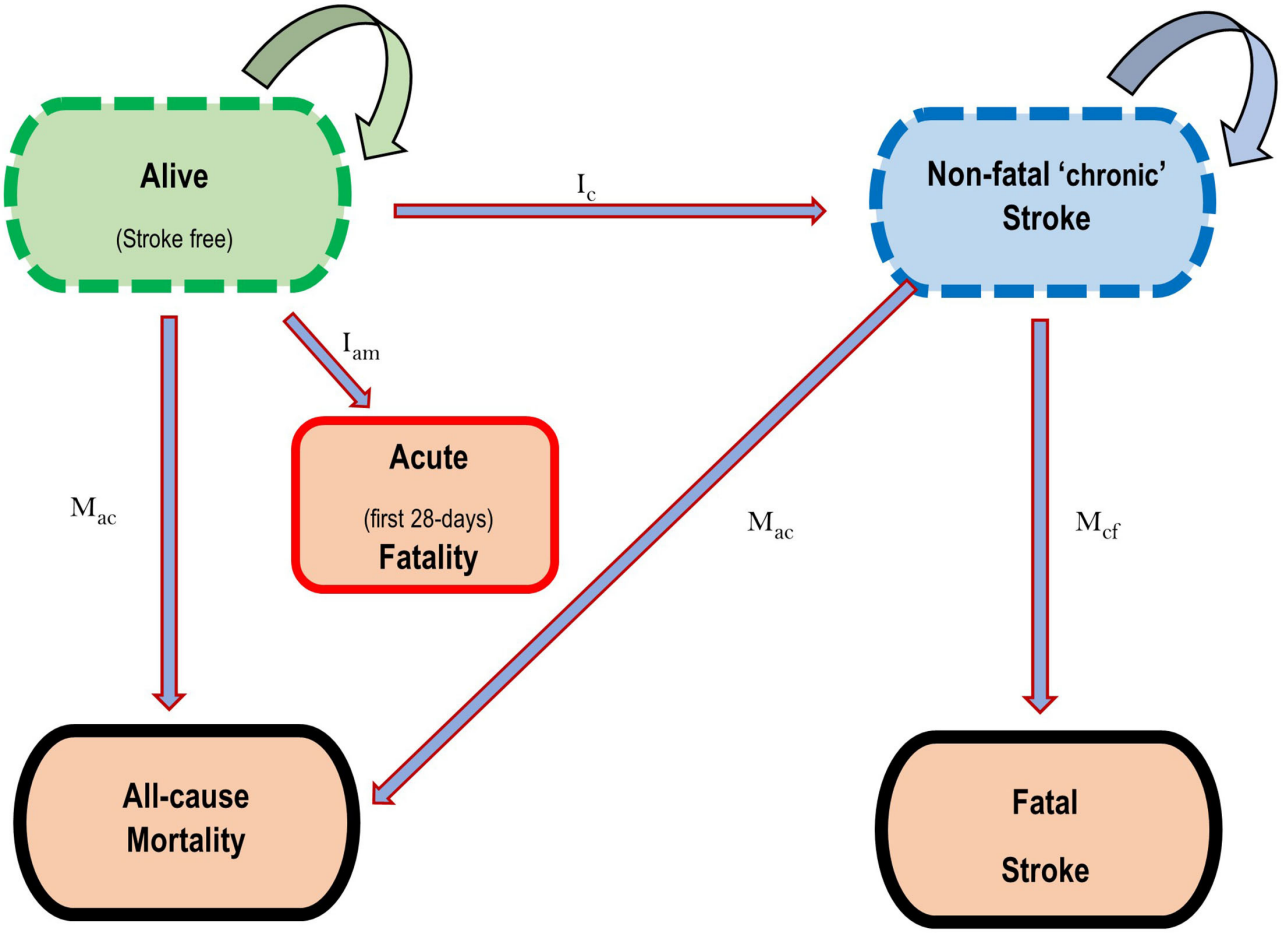
State transition diagram depicting the five health states for the Stroke Markov model. **I**_**c**_ = Incidence of Stroke in the first year, **I**_an_ = Case fatality in the first 28 days following incident CVD, **M**_*cf*_ = Case fatality in the first year for survivors of first 28-days Stroke mortality, **M**_*ac*_ = Mortality from all causes in that year. Straight arrows represent directions of movement of proportions of the population between health states, while circular arrows represent the probability of remaining in each health state. Death is an absorbing state.

For this study, the two major forms of stroke—ischaemic and haemorrhagic, were modelled. The state-transition Markov model shown in Figure 1, demonstrates the health states considered for each of the diseases. Five health states were considered: *Healthy* (alive without stroke), *Acute death* (dead within the first 28-days of a stroke event), *Diseased* (alive with “chronic” stroke, i.e., survivors of acute stroke death), *Dead from disease* (death from “chronic” stroke, i.e., stroke death among survivors of acute death) and *dead from other causes*. Transition hazards such as incidence, 28-day case fatality, case fatality (excess mortality) in month 2 to 12, and all-cause mortality influenced the movement of proportions of the population between these five health states, with death being an absorbing state. The DISMOD II software was used to derive other epidemiological parameters like case fatality that are seldomly reported, while maintaining consistency in the data (24).

The PMSLT Markov model has two identical populations stratified by sex and age. It has an “intervention” population that receives the intervention and a reference population that continues with current risk exposures as usual. As previously described, shifts in the risk factor distribution due to the PIF result in new disease incidence. This change in incidence leads to changes in prevalence and then mortality. The resulting disease mortality then feeds into the lifetable altering overall mortality rates and explicitly calculating life years. To obtain health-adjusted life years (HALYs), the years of life are adjusted for the poor quality of life due to the disease (in this case, stroke) being modelled and comorbidity using weights from the GBD (18). The PMSLT simulates these age-group and sex cohorts of the population until everyone dies or reaches the age of 100 years. Healthcare costs due to disease prevented are explicitly modelled over the respective time-horizons. In this study, costs were linked to each incident stroke, as our healthcare cost estimates pertained to the cost of care for first-ever stroke event. The differences in health outcomes (changes in blood pressure, stroke incidence, deaths, and HALYs) and healthcare costs between the two identical populations reflects the impact of the intervention. Health and economic outcomes were discounted at 3%.

### Scenario and Uncertainty Analysis

**Figure 2:**
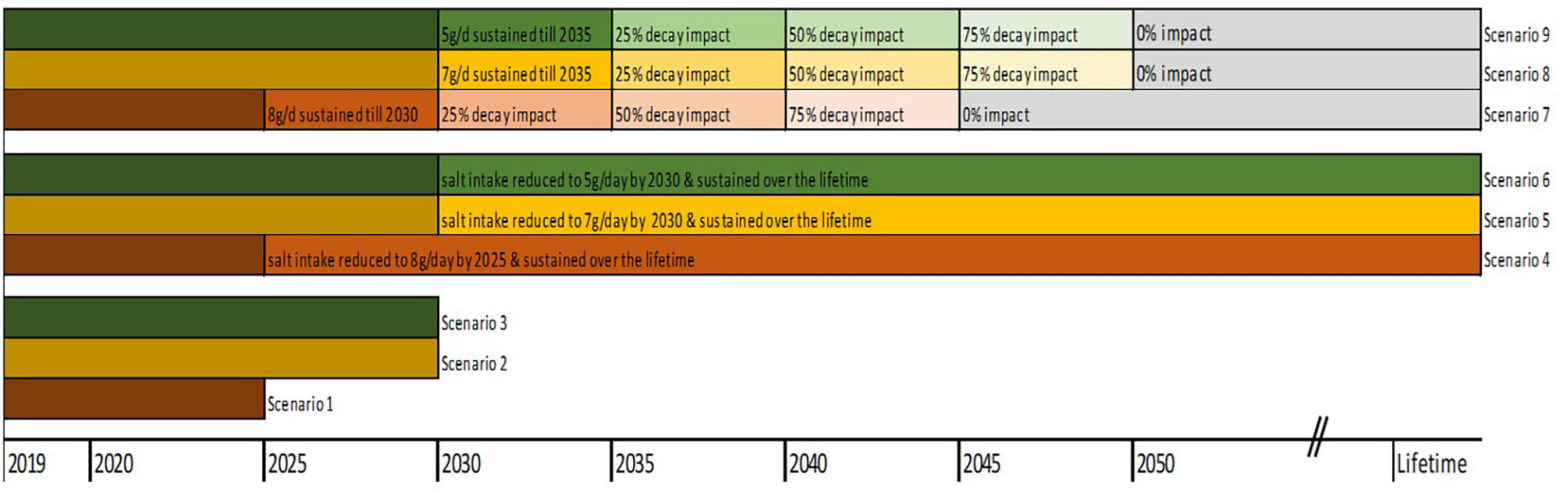
Baseline and scenario analyses of salt reduction targets.

The base case analysis assessed the changes in health and economic outcomes if everyone reduced their salt to 8 g/ day by 2025 (scenario 1) and 7 g/day by 2030 (scenario 2), both discounted at 3% (25). In a third scenario, we modelled the impact of a gradual reduction of salt intake down to 5 g/day (WHO target) by 2030 (scenario 3). For sensitivity analyses, we modelled longer time horizons, i.e., gradual reduction to 8 g/day to 2025 and sustained over the remaining lifetime (scenario 4); gradual reduction to 7 g/day by 2030 and sustained over the remaining lifetime (scenario 5); gradual reduction to 5 g/day by 2030 and sustained over the remaining lifetime (scenario 6). Analyses were also rerun at varying rates of intervention decay, i.e., (i) scenario 4 above with full impact sustained for 5 years and decline to 25% 5-yearly thereafter down to zero impact beyond the year 2045 (scenario 7); (ii) scenario 5 with full impact in first 5 years, and declining by 25% every 5 years down to zero impact by 2050 (scenario 8); (iii) scenario 6 with full impact in first 5 years, then decline by 25% 5 yearly down to 0% beyond year 2050 (scenario 9). The Figure 2 below depicts the modelled scenarios.

For parameter estimates with uncertainty, we applied the following distributions: normal (salt- BP relationship), lognormal (relative risks), and gamma (healthcare costs). Parameters were drawn randomly from these distributions in probabilistic sensitivity analyses implemented using the Ersatz software (26), with 2,000 simulations executed.

## Results

### Estimated Effects on Blood Pressure and Hypertension

With 2019 as baseline year, our model estimates that a gradual reduction (on average, 0.383 and 0.033 g annually for 30–49-year-old men and women, respectively) of salt consumption to 8/day by 2025 could reduce mean SBP by 1.9 and 0.12 mmHg in men and women, respectively. This translates to a reduction in hypertension prevalence of 2.3% (671,000 men) and 0.2% (68,000 women) by 2025. Furthermore, gradual reductions to 7 g/day (on average, 0.300 and 0.109 g/day annually for 30–49-year-old men and women, respectively) by 2030 could lead to 927,000 (3.1%) fewer men and 284,000 (0.9%) fewer women with hypertension. If salt consumption were to go down to 5 g/day (that is, annual reductions of 0.482 and 0.291 g/day for 30–49-year-old men and women, respectively) by 2030, mean SBP could reduce by about 4.0 mmHg for men and 2.2 mmHg for women, translating to over 1.4 million (4.9%) fewer men and 726,000 (2.0%) fewer women with hypertension (Table 1).

**Table 1:** Estimated reductions in mean systolic blood pressure, the proportion and number of people with hypertension in Vietnam.

	**Men**			**Women**			**Both**		
	**Mean (95%UI), mmHg**	**Prevalence, % (95%UI)**	**Number In 000s (95%UI)**	**Mean (95%UI), mmHg**	**Prevalence, % (95%UI)**	**Number In 000s (95%UI)**	**Mean (95%UI), mmHg**	**Prevalence, % (95%UI)**	**Number, in 000s (95%UI)**
Scenario 1	1.9	2.3	671	0.1	0.2	68	1.0	1.2	739
	(1.4 to 2.7)	(0.8 to 4.5)	(222 to 1,332)	(0.1 to 0.2)	(0.0 to 0.6)	(2 to 205)	(0.8 to 1.4)	(0.5 to 2.3)	(282 to 1,402)
Scenario 2	2.7	3.1	927	0.7	0.9	284	1.7	2.0	1,211
	(1.9 to 3.8)	(1.0 to 6.3)	(287 to 1,867)	(0.6 to 1.2)	(0.2 to 2.1)	(55 to 651)	(1.3 to 2.4)	(0.8 to 3.6)	(501 to 2,178)
Scenario 3	4.0	4.9	1,432	2.2	2.3	726	3.2	3.5	2,158
	(3.1 to 5.9)	(1.3 to 9.8)	(395 to 2,894)	(1.7 to 3.3)	(0.5 to 5.3)	(142 to 1,674)	(2.4 to 4.3)	(1.5 to 6.3)	(886 to 3,872)

*Scenario 1: Gradual reduction in salt intake to 8 g/day between 2019 and 2025; Scenario 2: Gradual reduction in salt intake to 7 g/day between 2019 and 2030; Scenario 3: Gradual reduction in salt intake to 5 g/day (WHO target) between 2019 and 2030. UI, uncertainty interval*.

### Projected Changes in Stroke Incidence

**Figure 3:**
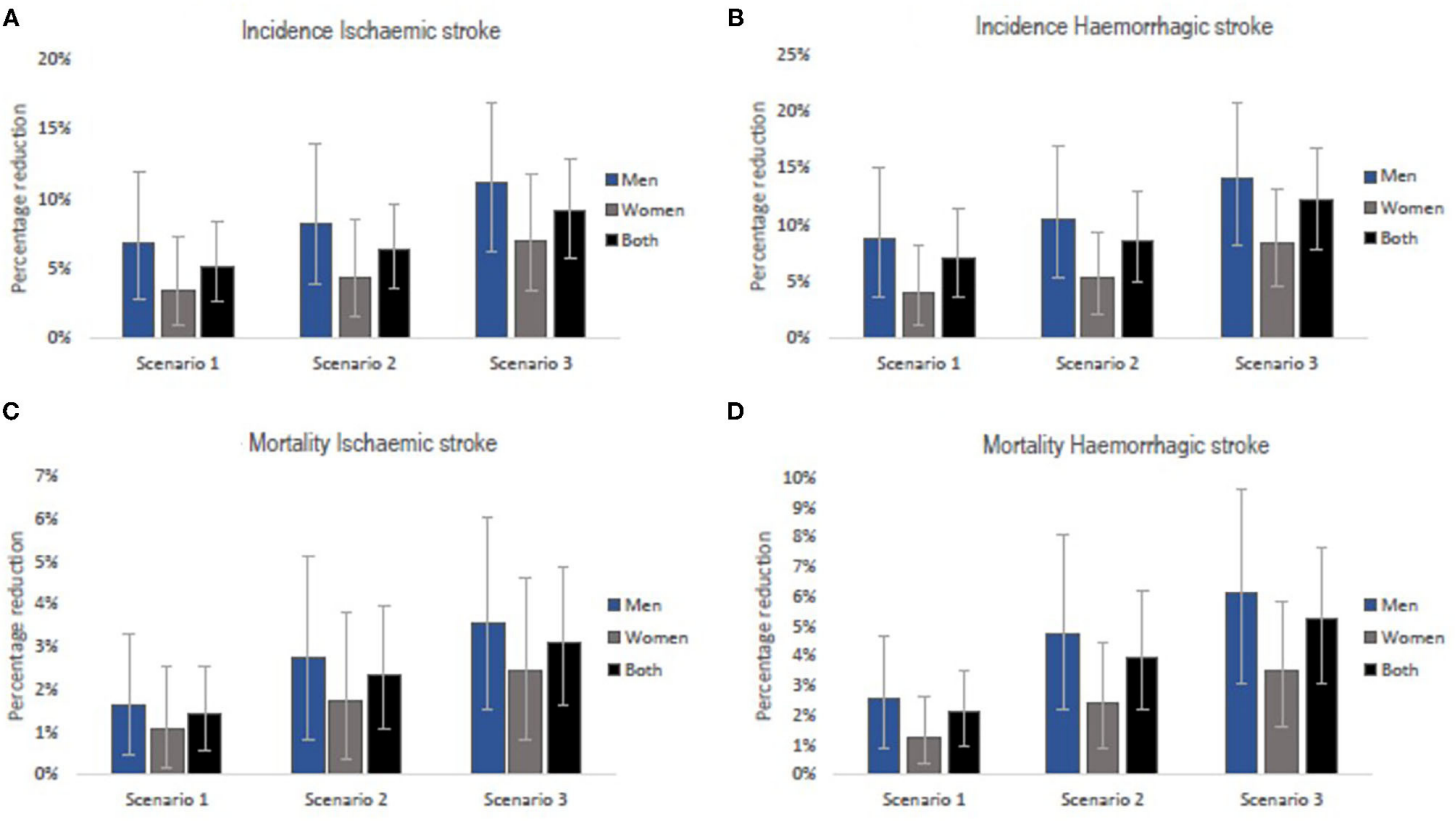
Relative reductions in stroke incidence rates **(A,B)** and mortality rates **(C,D)** if the national (Scenarios 1 and 2) and WHO (Scenario 3) salt targets were achieved in Vietnam. The bars are the best estimates while the whiskers are the 95% uncertainty intervals, i.e., 2.5 and 97.5 percentiles.

A gradual reduction in salt intake down to 8 g/day by 2025 could cumulatively avert 38,422 (95% uncertainty interval [UI]: 19,265–62,531) new cases of ischaemic stroke and 41,592 (95%UI: 21,063–66,829) new cases of haemorrhagic stroke in Vietnam. In addition, a gradual reduction in salt intake down to 7 g/day by 2030 could avert 87,955 (95% UI: 48,385–133,216) incident cases of ischaemic stroke and 92,816 (95% UI: 53,903–139,931) incident haemorrhagic strokes. If the WHO target of 5 g/day were achieved by 2030, there could be 127,522 (95% UI: 79,694–179,775) fewer new cases of ischaemic stroke and 130,150 (95% UI: 82,918–180,542) fewer new cases of haemorrhagic stroke (Table 2). In all three scenarios, reductions in incident strokes were greater in men compared to women (Scenario 1: 6.8% vs. 3.4% for ischaemic and 8.7% vs. 4.0% for haemorrhagic stroke; Scenario 2: 8.2% vs. 4.4% for ischaemic and 10.5% vs. 5.2% for haemorrhagic stroke; Scenario 3: 11.2% vs. 7.0% for ischaemic and 14.1% vs. 8.4% for haemorrhagic stroke (Figure 3).

**Table 2:** Absolute reductions in incident strokes among adults in Vietnam from 2019 to 2030 and over the lifetime.

	**Ischaemic stroke**			**Haemorrhagic stroke**		
**Base analysis**	**Men *N* (95% UI)**	**Women *N* (95% UI)**	**Both *N* (95% UI)**	**Men *N* (95% UI)**	**Women *N* (95% UI)**	**Both *N* (95% UI)**
Scenario 1	26,026	12,396	38,422	33,455	8,136	41,592
	(10,832–45,967)	(3,147–26,644)	(19,265–62,531)	(13,535–58,264)	(2,385–16,453)	(21,063–66,829)
Scenario 2	58,250	29,705	87,955	73,469	19,348	92,816
	(26,879–98,940)	(10,496–57,787)	(48,385–133,216)	(37,160–119,247)	(7,637–34,747)	(53,903–139,931)
Scenario 3	79,767	47,755	127,522	99,031	31,118	130,150
	(43,483–119,706)	(22,895–79,900)	(79,694–179,775)	(57,496–146,539)	(16,561–49,017)	(82,918–180,542)
**Sensitivity analysis**						
Scenario 4	281,789	115,477	397,267	297,655	75,674	373,328
	(122,584–474,551)	(25,682–250,683)	(206,737–623,454)	(138,099–473,912)	(17,456–157,881)	(200,838–558,219)
Scenario 5	347,259	179,595	526,854	360,760	116,356	477,116
	(173,340–542,156)	(62,988–321,514)	(308,774–757,595)	(192,170–544,478)	(42,151–200,810)	(284,050–670,203)
Scenario 6	525,533	361,890	887,424	536,155	232,423	768,577
	(318,991–760,985)	(195,316–555,027)	(597,364–1,185,196)	(341,998–746,822)	(124,758–350,976)	(526,175–1,019,372)
Scenario 7	108,021	48,833	156,854	144,293	33,594	177,887
	(39,548–188,715)	(10,682–103,480)	(74,052–253,348)	(63,773–235,470)	(9,907–64,083)	(92,147–271,777)
Scenario 8	159,725	77,094	236,819	201,038	52,323	253,361
	(71,840–260,121)	(20,222–151,785)	(133,798–360,652)	(107,628–312,018)	(18,106–94,975)	(154,421–366,041)
Scenario 9	221,723	129,746	351,469	279,785	87,231	367,016
	(119,686–336,699)	(54,903–221,746)	(210,881–493,588)	(167,125–397,474)	(41,393–141,495)	(241,485–504,246)

*Scenario 1 = Gradual reduction in salt intake to 8 g/day between 2019 and 2025; Scenario 2 = Gradual reduction in salt intake to 7 g/day between 2019 and 2030; Scenario 3 = Gradual reduction in salt intake to 5 g/day (WHO target) between 2019 and 2030; Scenario 4 = Scenario 1 achieved and sustained for the remaining lifetime; Scenario 5 = Scenario 2 achieved and sustained for the remaining lifetime; Scenario 6 = Scenario 3 achieved and sustained for the remaining lifetime; Scenario 7 = Scenario 1 achieved and effect gradually phases out over 20 years with zero effect beyond 2045; Scenario 8 = Scenario 2 achieved and effect gradually phases out over 20 years with zero effect beyond 2050; Scenario 9 = Scenario 3 achieved and effect gradually phases out over 20 years with zero effect beyond 2050; N, Number; UI, uncertainty interval*.

### Projected Changes in Stroke Mortality

Our model projects that a gradual reduction of population salt intake to 8 g/day by 2025, could lead to 3,663 (95% UI: 1,415–6,568) fewer deaths (1.4% reduction) from ischaemic stroke and 14,498 (95% UI: 6,701–24,304) fewer deaths (2.1% reduction) from haemorrhagic stroke in Vietnam. If the current salt consumption were gradually reduced to 7 g/day by 2030, there could be 13,030 (95% UI: 6,060–22,023) and 42,227 (95% UI: 23,259–66,480) deaths avoided from ischaemic (2.3% reduction) and haemorrhagic (4.0% reduction) stroke, respectively. A larger number of fatal events, that is, 17,249 (95% UI: 9,048–27,155) for ischaemic stroke (3.1% reduction) and 56,209 (33,117–81,734) for haemorrhagic stroke (5.3% reduction) could be avoided if average population salt consumption were reduced to 5 g/day by 2030 (Table 3). The estimated reductions in stroke mortality were greater for men than women (Scenario 1: 1.7% vs. 1.1% for ischaemic and 2.5% vs. 1.3% for haemorrhagic; Scenario 2: 2.8% vs. 1.7% for ischaemic and 4.8% vs. 2.4% for haemorrhagic; Scenario 3: 3.5% vs. 2.4% for ischaemic and 6.2% vs. 3.5% for haemorrhagic (Figure 3).

**Table 3:** Absolute reductions in stroke mortality among adults in Vietnam from 2019 to 2030 and over the lifetime.

	**Ischaemic stroke**			**Haemorrhagic stroke**		
**Base analysis**	**Men *N* (95% UI)**	**Women *N* (95% UI)**	**Both *N* (95% UI)**	**Men *N* (95% UI)**	**Women *N* (95% UI)**	**Both *N* (95% UI)**
Scenario 1	2,521	1,143	3,663	11,481	3,017	14,498
	(729–5,066)	(153–2,680)	(1,415–6,568)	(3,925–21,329)	(829–6,179)	(6,701–24,304)
Scenario 2	9,072	3,959	13,030	33,415	8,812	42,227
	(2,724–16,913)	(844–8,674)	(6,060–22,023)	(15,265–57,021)	(3,301–16,253)	(23,259–66,480)
Scenario 3	11,711	5,539	17,249	43,243	12,966	56,209
	(5,104–19,939)	(1,819–10,506)	(9,048–27,155)	(21,624–67,494)	(5,883–21,370)	(33,117–81,734)
**Sensitivity analysis**						
Scenario 4	158,035	63,995	222,030	205,366	47,683	253,049
	(68,239–266,488)	(14,159–139,303)	(115,129–349,135)	(94,262–326,976)	(11,187–99,615)	(135,607–376,978)
Scenario 5	194,797	99,470	294,267	247,904	72,883	320,787
	(96,741–304,265)	(34,636–178,368)	(171,682–424,978)	(131,185–372,313)	(26,701–125,848)	(189,592–450,226)
Scenario 6	295,239	200,407	495,646	367,211	144,764	511,975
	(178,923–428,698)	(107,210–308,078)	(333,325–661,912)	(235,595–510,946)	(78,511–218,538)	(350,943–680,534)
Scenario 7	60,890	27,515	88,405	105,379	23,002	128,381
	(22,583–106,855)	(5,994–58,867)	(41,507–143,607)	(46,826–172,341)	(6,753–43,922)	(66,205–197,377)
Scenario 8	90,018	43,447	133,464	145,248	35,431	180,679
	(40,740–146,906)	(11,373–85,822)	(75,076–203,699)	(77,719–225,633)	(12,535–63,619)	(107,685–261,116)
Scenario 9	125,126	73,157	198,283	202,390	59,191	261,581
	(67,057–190,961)	(30,995–125,015)	(118,213–279,685)	(121,054–287,690)	(27,451–95,818)	(173,639–358,635)

*Scenario 1 = Gradual reduction in salt intake to 8 g/day between 2019 and 2025; Scenario 2 = Gradual reduction in salt intake to 7 g/day between 2019 and 2030; Scenario 3 = Gradual reduction in salt intake to 5 g/day (WHO target) between 2019 and 2030; Scenario 4 = Scenario 1 achieved and sustained for the remaining lifetime; Scenario 5 = Scenario 2 achieved and sustained for the remaining lifetime; Scenario 6 = Scenario 3 achieved and sustained for the remaining lifetime; Scenario 7 = Scenario 1 achieved and effect gradually phases out over 20 years with zero effect beyond 2045; Scenario 8 = Scenario 2 achieved and effect gradually phases out over 20 years with zero effect beyond 2050; Scenario 9 = Scenario 3 achieved and effect gradually phases out over 20 years with zero effect beyond 2050; N, Number; UI, uncertainty interval*.

### Health-Adjusted Life Years and Healthcare Costs

**Figure 4:**
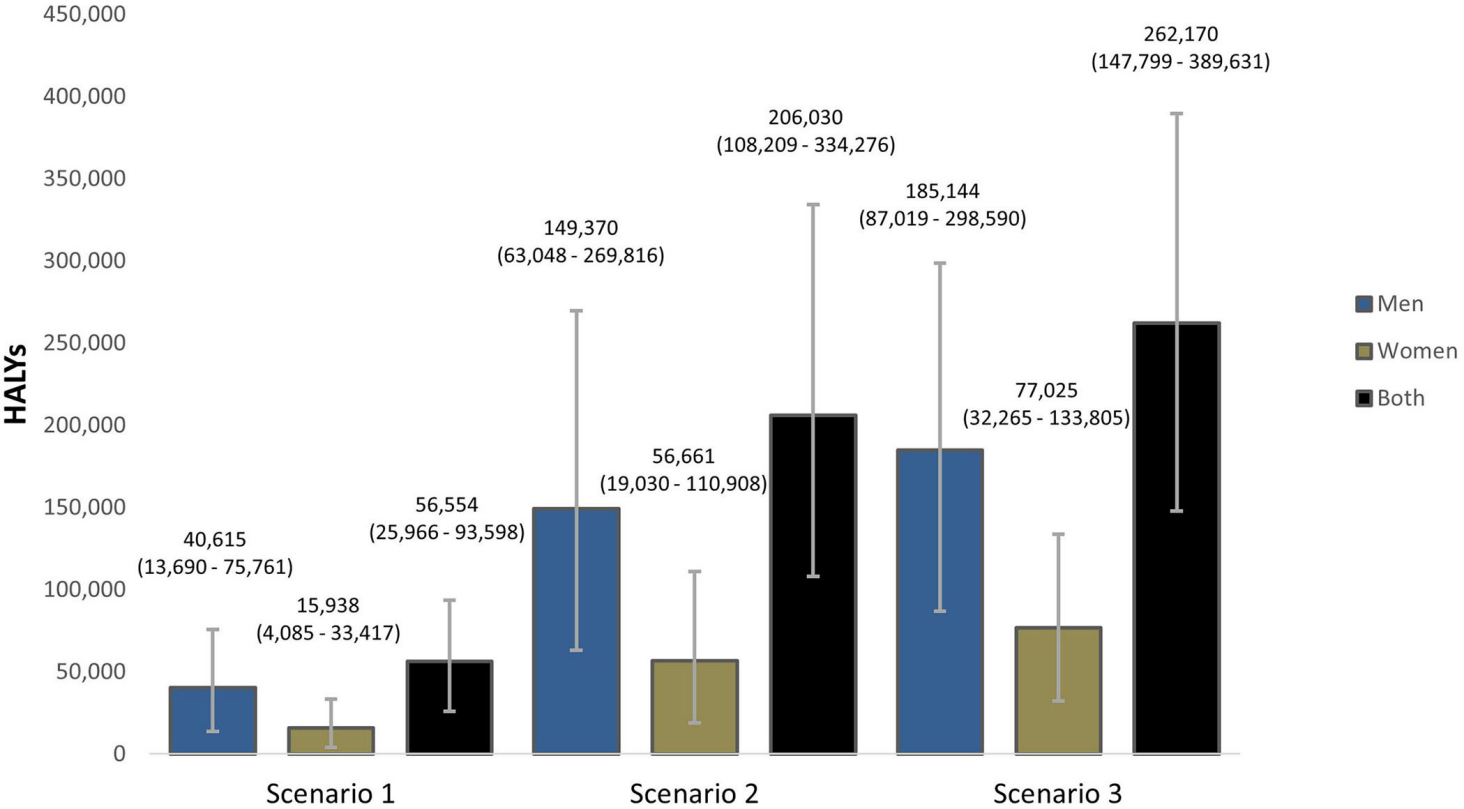
Projected Health-adjusted life years (HALYs) that could be gained if the national (Scenarios 1 and 2) and WHO (Scenario 3) salt targets were achieved in Vietnam. The bars are the best estimates while the whiskers are the 95% uncertainty intervals, i.e., 2.5 and 97.5 percentiles.

Overall, a gradual reduction in dietary salt intake to 8 g/day by 2025 could result in 56,554 (95% UI: 25,966–93,598) health-adjusted life years (HALY) gained while reductions to 7 g/day by 2030 could result in 206,030 (95% UI: 108,209–334,276) HALYs gained. Furthermore, our model projects that gradual reductions to 5 g/day by 2030 could gain 262,170 (95% UI: 147,799–389,631) stroke-related HALYs in Vietnam (Figure 4).

Averted new cases of stroke are projected to result in savings in healthcare costs. Scenario 1 was projected to save US$ 18.5 (95% UI: 4.3–43.1) million cumulatively for first ever ischaemic stroke and US$ 24.1 (95% UI: 7.0–58.5) million for first ever haemorrhagic stroke treatments by 2025. Scenario 2 was projected to save US$ 38.8 (13.0–85.7) million for ischaemic stroke and US$ 49.3 (15.3–110.3) million for haemorrhagic stroke while scenario 3 could lead to savings of US$ 55.7 million and US$ 66.6 million for first ever ischaemic and haemorrhagic stroke treatments cumulatively by 2030 (Table 4).

**Table 4:** Projected healthcare costs saved from avoided incident strokes among adults in Vietnam from 2019 to 2030 and over the lifetime.

	**Ischaemic stroke**			**Haemorrhagic stroke**		
**Base analysis**	**Men Mean (95% UI)**	**Women Mean (95% UI)**	**Both Mean (95% UI)**	**Men Mean (95% UI)**	**Women Mean (95% UI)**	**Both Mean (95% UI)**
*In millions, US$*						
Scenario 1	12.4	6.0	18.5	19.3	4.8	24.1
	(3.1 to 33.6)	(0.9 to 18.7)	(5.7 to 43.1)	(4.8 to 50.7)	(0.7 to 16.1)	(7.0 to 58.5)
Scenario 2	25.4	13.4	38.8	39.2	10.2	49.3
	(6.9 to 62.5)	(2.5 to 36.3)	(13.0 to 85.7)	(10.3 to 94.2)	(2.2 to 25.5)	(15.3 to 110.3)
Scenario 3	34.9	20.8	55.7	50.6	16.0	66.6
	(10.5 to 78.8)	(5.8 to 49.8)	(20.3 to 113.8)	(15.9 to 111.1)	(5.0 to 37.9)	(24.3 to 138.3)
**Sensitivity analyses**						
***In millions, US$***						
Scenario 4	79.3	30.6	109.9	105.8	23.8	129.6
	(22.9 to 175.9)	(5.3 to 86.4)	(38.7 to 234.6)	(26.6 to 260.6)	(4.1 to 68.8)	(37.4 to 294.8)
Scenario 5	93.6	46.4	140.0	123.6	35.9	159.5
	(29.3 to −199.7)	(9.7 to 115.2)	(51.9 to 290.6)	(34.9 to 26.8)	(7.9 to 86.0)	(50.1 to 337.2)
Scenario 6	137.9	88.8	226.7	180.9	67.4	248.4
	(53.1 to 277.7)	(31.4 to 186.7)	(95.8 to 440.9)	(60.4 to 379.2)	(23.3 to 134.4)	(97.4 to 505.4)
Scenario 7	44.7	19.4	64.1	66.5	15.3	81.8
	(10.6 to 106.4)	(3.0 to 57.8)	(21.6 to 144.6)	(15.6 to 165.0)	(2.8 to 43.6)	(22.9 to 187.1)
Scenario 8	60.6	28.2	88.7	86.9	22.3	109.2
	(18.1 to 145.2)	(5.1 to 80.9)	(32.4 to 194.2)	(25.3 to 201.9)	(4.4 to 60.3)	(35.6 to 238.3)
Scenario 9	82.3	47.5	129.8	119.1	37.5	156.6
	(26.9 to 176.2)	(11.9 to 113.0)	(50.9 to 257.9)	(36.9 to 270.8)	(11.6 to 89.0)	(55.7 to 337.5)

*Scenario 1 = Gradual reduction in salt intake to 8 g/day between 2019 and 2025; Scenario 2 = Gradual reduction in salt intake to 7 g/day between 2019 and 2030; Scenario 3 = Gradual reduction in salt intake to 5 g/day (WHO target) between 2019 and 2030; Scenario 4 = Scenario 1 achieved and sustained for the remaining lifetime; Scenario 5 = Scenario 2 achieved and sustained for the remaining lifetime; Scenario 6 = Scenario 3 achieved and sustained for the remaining lifetime; Scenario 7 = Scenario 1 achieved and effect gradually phases out over 20 years with zero effect beyond 2045; Scenario 8 = Scenario 2 achieved and effect gradually phases out over 20 years with zero effect beyond 2050; Scenario 9 = Scenario 3 achieved and effect gradually phases out over 20 years with zero effect beyond 2050; UI, uncertainty interval*.

### Sensitivity Analyses

In addition to the 6 years (2019–2025) and 11 years (2019–2030) time horizons in the base analyses, six additional scenarios were run with lifetime horizons. As expected, for scenarios 4 to 6 in which the 2025 and 2030 salt target levels were sustained for the remaining lifetime, larger impacts were observed ranging from about 771,000 to 1.6 million cumulative new cases of both forms of stroke and about 475,000 to over a million cumulative stroke deaths averted. For scenarios 7 to 9, in which we gradually phased out the intervention effect with zero impact beyond 20- and 25-years post target achievement, health gains were almost halved compared to the sustained scenarios, but these were still about 2 to 4 times the benefits observed in the base analysis. Similar patterns were observed in for HALYs and healthcare cost offsets (Tables 2, 3 and Supplementary File).

## Discussion

We evaluated the potential impact of attaining three population dietary salt reduction targets stipulated in the Vietnam national and WHO recommendations. Our model projects that through modest reductions in mean systolic blood pressure, gradual reductions in average population salt consumption could avert between 80,000 to 250,000 non-fatal and 18,000 to 73,000 fatal strokes over six- to 11-year time horizons. Furthermore, there were large gains in healthy life years and savings in acute stroke direct health expenditures. These benefits were substantially larger (~6 times baseline and ~28 times baseline scenarios for incidence and mortality, respectively) if sustained over the lifetime. However, these lifetime impacts were reduced (correspondingly ~3 times to ~11 times the baseline scenarios) if changes were not maintained. Compared to women, men were the bigger beneficiaries of these impacts.

### Comparisons and Implications of the Findings

In a previous modelling study evaluating a suite of interventions for CVD prevention in Vietnam (27), authors found that reducing population salt intake via media campaign could avert 45,939 DALYs annually. Results from our base analysis ranged from 9,425 to 23,833 HALYs annually. On par value, this could appear lower than the Ha *et al*. findings. However, our HALYs are essentially stroke-related, as opposed to the former study which included ischaemic heart disease and stroke. It is also possible that disparities in modelling methods (28) might explain some of these differences. Basu et al. modelled the impact of a 3 g/day reduction in salt intake in India over a 30-year time horizon (29). They found that such an intervention could reduce stroke incidence rates by 9–13%, which appears on par with our work. As in our study, research from Brazil (30) and Cameroon (31) has similarly modelled the potential impact of attaining the WHO target of 5 g/day of salt intake, and equally found large reductions in CVD events and premature mortality (31, 32). Consistent with previous studies (14, 21), benefits were greater in men than in women. Evidence has shown that the impact of salt reduction increases linearly with blood pressure, with a steeper gradient in those with mean SBP > 140 mmHg and those with higher baseline salt intake (9). The Vietnam 2015 STEPS survey used in our analysis showed that men had higher average salt consumption levels and higher mean blood pressures. It is therefore unsurprising that they were more likely to accrue greater gains than the women. Given that we assume salt reduction is proportional to current consumption, hence the higher consumption in men translated to comparatively larger absolute reductions in salt intake required to meet the national targets, and therefore more gains in health impacts.

Our model projects slightly larger reductions in incidence of and mortality from haemorrhagic stroke compared to ischaemic stroke. Substantial evidence exists on the link between blood pressure and risk of stroke, demonstrating greater relative risk of haemorrhagic stroke per unit increase in blood pressure compared to ischaemic stroke (33, 34). We modelled the impact of salt reduction on stroke in a two-step process, that is, mediated via blood pressure. As a result, reduction in average population blood pressure is likely to lead to slightly bigger reductions in incidence of haemorrhagic stroke compared to ischaemic stroke. In addition, data from stroke units in Vietnam used in our study showed that acute case fatality rates in the first 28 days were higher (almost double for some age groups) for people with haemorrhagic stroke compared to those with ischaemic stroke (19). These disparities in risk of and death from either form of stroke in part might explain the slightly larger reductions in incidence and deaths from haemorrhagic stroke observed in our projections.

There are ongoing concerns about potential negative side-effects of whole of populations reduction in salt intake down to 5–6 g/day, citing insufficient evidence (35) and potential unintended consequences to hormones (increased renin, adrenaline, aldosterone) and lipids (36). Most of these concerns are based on observational studies with less robust methods and short-term trials of large salt reductions (37). Evidence from methodologically robust studies with modest long-term reduction in salt intake has shown consistent benefits of low salt consumption on blood pressure and CVD (9, 38), thereby supporting current global recommendations of low sodium diets (11, 39, 40). With current salt consumption in Vietnam more than twice these recommendations, very high rates of stroke, and the substantial health and economic gains shown in this study, there is a strong case for population-wide reductions in salt consumption.

From a policy perspective, the accurate quantification and projections of such diet-related indicators play a critical role in developing NCD strategies and policies. It is important to acknowledge that strong national NCD policies need to match the severity of the current and future burden of NCDs. By so doing guide government action on the most critical dietary factors driving population health, as well the population groups at risk. While Vietnam has recognised the importance of addressing NCDs, there has been little rigorous analyses evaluating policies, strategies or programs monitoring the control of NCDs, particularly for salt consumption and hypertension. More so, despite strong government support for NCD prevention and control at national level, implementation of NCD programs has been lagging. Our health and economic projections therefore present an important contribution filling a critical policy-relevant knowledge gap. In addition, we hope this will set the stage for future work to understand the challenges around program implementation and guide development of innovative solutions towards improving NCD prevention and control.

### Strengths and Limitations

This study has a number of strengths. First, we used local data on dietary salt consumption and blood pressure distribution obtained from the NCD surveillance WHO-STEPS study, which is a nationally representative survey. Second, our analysis accounts for the differential impact of salt reduction on blood pressure by age, sex, and hypertension status, which enhances the accuracy of our estimates. Third, by quantitatively estimating the potential health and economic impact of national public health strategies under different time horizons as well as their uncertainty, these findings have high policy-relevance. This represents an advancement from the limited evidence from Vietnam.

Regarding limitations, first, our model is a closed cohort model, which in its current form does not allow for new births and migration. Hence, our results are likely conservative as they do not capture the benefits of younger cohorts as they age into the future. In addition, other blood pressure-related outcomes such as ischaemic heart disease, hypertensive heart disease and chronic kidney disease were not included, as these were not the focus of this work. As a result, the benefits of salt reduction are likely to be considerably larger than presented here. Thirdly, data on healthcare costs are limited to first-time hospitalisation for acute stroke and do not capture other healthcare costs that accrue post-acute hospitalisation such as rehabilitation, follow-up visits and medication. In addition, wider societal costs like carer responsibilities and productivity losses are not included, thereby underestimating the costs. Future studies should consider wider perspectives to capture these costs. Fourth, the healthcare cost data was obtained from a single tertiary centre. This may not be representative of the costs of care obtained at other peripheral hospitals. It may also overestimate costs for patients treated in primary care settings. Moreover, healthcare costs from other conditions that accrue because of people living longer from the intervention are not included. While the balance of the impact these limitations is not immediately clear, other studies incorporating these costs demonstrated net cost savings (14, 21).

In conclusion, this modelling study shows that modest reductions in population blood pressure via achieving the Vietnam national and WHO salt reduction targets could lead to substantial benefits—gains in healthy life years, reducing health expenditures, avoiding CVD events and associated misery. There is urgent need for specific salt reduction strategies in Vietnam to curb the current and impending CVD and economic burden. Future studies that account for the equity and budget impacts of such strategies would be informative for policy makers as they grapple with implementation.

## Data Availability Statement

The original contributions presented in the study are included in the article/Supplementary Material, further inquiries can be directed to the corresponding author/s.

## Author Contributions

LA: conceptualisation, data curation, formal analysis, methodology, and writing—original draft. HP: conceptualisation, data curation, validation, and writing—review and editing. DP: conceptualisation, validation, and writing—review and editing. LC: methodology and writing—review and editing. JV: conceptualisation, methodology, validation, supervision, and writing—review and editing. All authors read and approved the final version of the paper.

## Conflict of Interest

The authors declare that the research was conducted in the absence of any commercial or financial relationships that could be construed as a potential conflict of interest.
