# Supplementary material for: Dietary Salt Reduction, Prevalence of Hypertension and Avoidable Burden of Stroke in Vietnam: Modelling the Health and Economic Impacts
Source: Front Public Health. 2021 Jun 4;9:682975. doi: 10.3389/fpubh.2021.682975 (PMC8213032; doi:10.3389/fpubh.2021.682975)
Supplement: Supplementary file 1 [file Data_Sheet_1.pdf]

Supplementary file

1. Input data and sources

Parameter	Values		Source
Mean systolic blood pressure [SBP] (mmHg)	Men<br>Mean (SD)	Women<br>Mean (SD)	Vietnam 2015 WHO STEPS survey [1]
18-29 years	118.0 (15.1)	106.1 (12.6)	
30-49 years	124.2 (21.5)	114.6 (21.3)	
50-69 years	134.3 (29.8)	128.8 (25.7)	
Mean salt consumption (g/day)	Men<br>Mean (SD)	Women<br>Mean (SD)	Vietnam 2015 WHO STEPS survey [1]
18-29 years	10.3 (2.4)	8.2 (2.3)	
30-49 years	10.7 (2.9)	8.6 (2.2)	
50-69 years	10.6 (2.7)	7.9 (2.4)	
Impact of salt (for every 4g salt) on SBP (mmHg)	Men<br>Mean (95%CI)	Women<br>Mean (95%CI)	He et al. 2013 [2]
Normotensive	2.42 (1.29 – 3.56)	2.42 (1.29 – 3.56)	
Hypertensive	5.39 (4.15 – 6.62)	5.39 (4.15 – 6.62)	
Costs of first-ever stroke treatment (in US$)	Men<br>Mean (SD)	Women<br>Mean (SD)	Pham TL. [3]
Ischaemic stroke	535 (514)	535 (514)	
Haemorrhagic stroke	637 (691)	637 (691)	
First 28-days case fatality rates	Men	Women	Pham TL. [3]
Ischaemic stroke			
< 45 years	3.4%	5.8%	
45-54 years	6.4%	4.2%	
55-64 years	5.3%	3.7%	
65-74 years	9.6%	9.1%	
75-84 years	8.4%	11.3%	
≥ 85 years	13.6%	22.3%	
Haemorrhagic stroke			
< 45 years	22.1%	18.2%	
45-54 years	22.5%	28.1%	
55-64 years	27.4%	35.4%	
65-74 years	21.6%	23.8%	
75-84 years	30.5%	36.4%	
≥ 85 years	38.5%	52.2%	
Relative risks for stroke due to increased SBP	Data by five-year age groups and sex are freely available in the GBD Results tool: http://ghdx.healthdata.org/gbd-results-tool		Global Burden of Disease study 2019 [4].
Overall mortality rates			
All-cause prevalent years lived with disability (pYLD) rates			
Stroke epidemiology (incidence, prevalence, mortality, disability) rates			

2. Additional results from scenario analyses

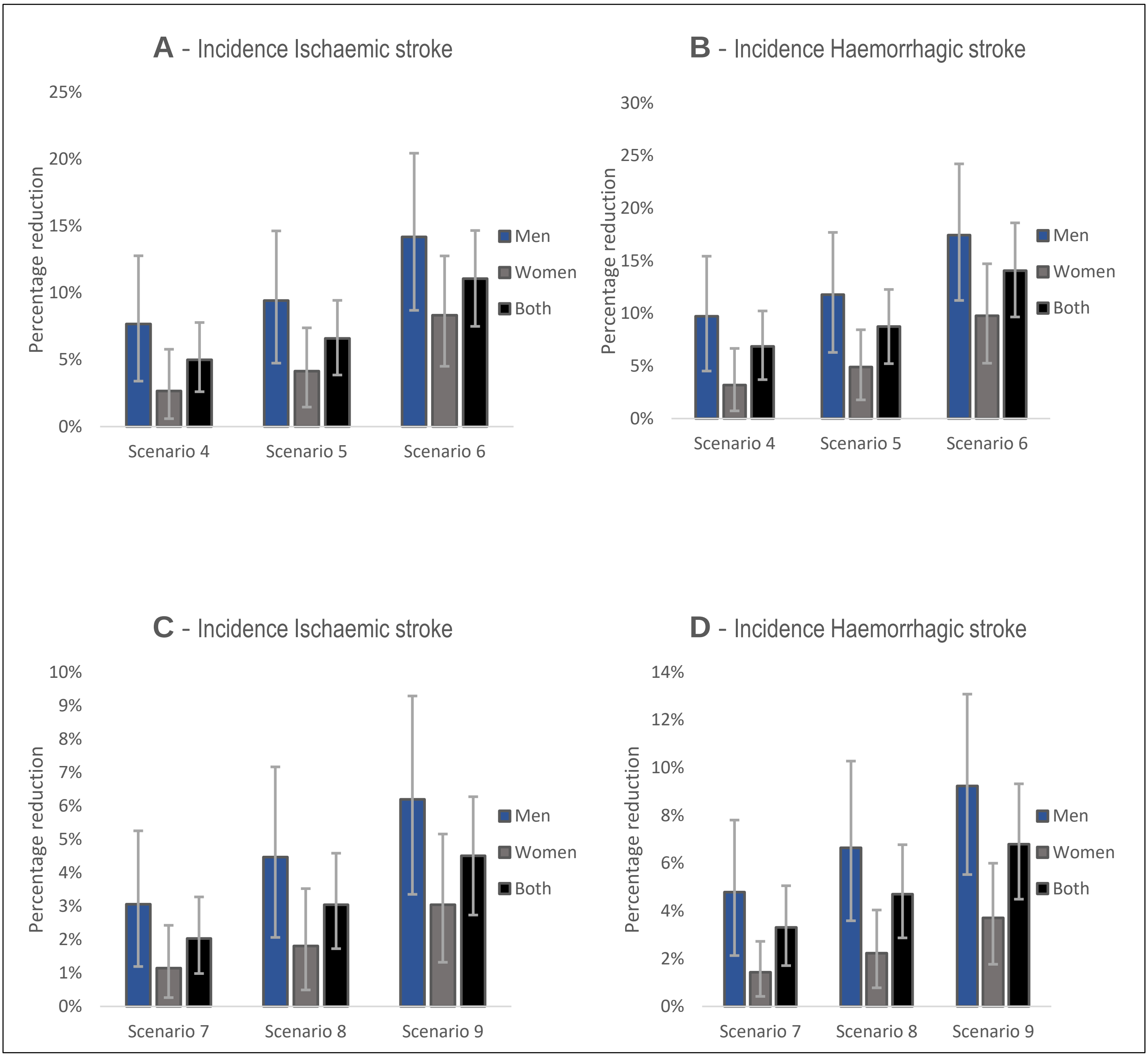

Figure 2: Relative reductions in cumulative stroke incidence rates over the lifetime under different scenarios

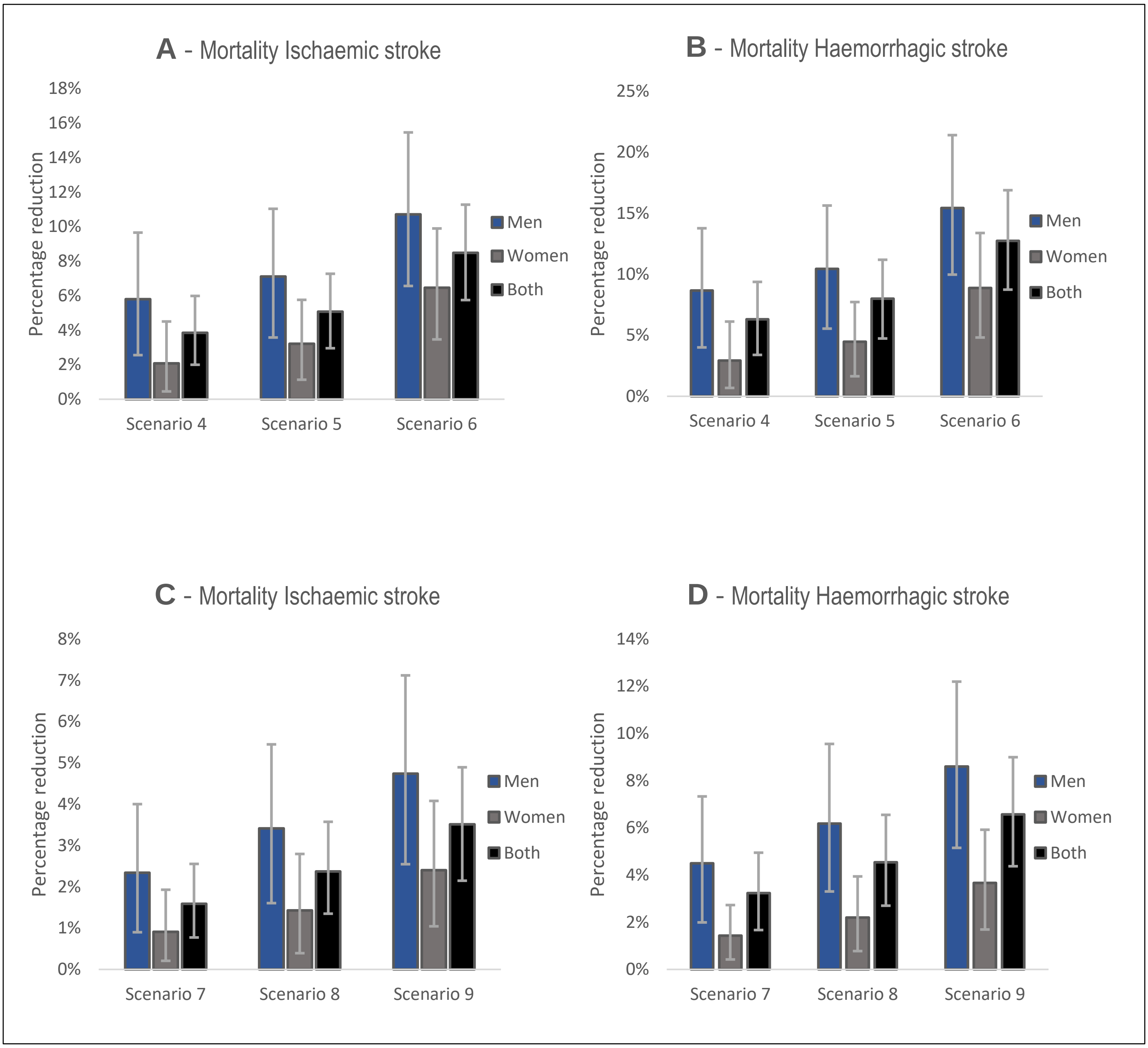

Figure 3: Relative reductions in cumulative stroke mortality rates over the lifetime under different scenarios

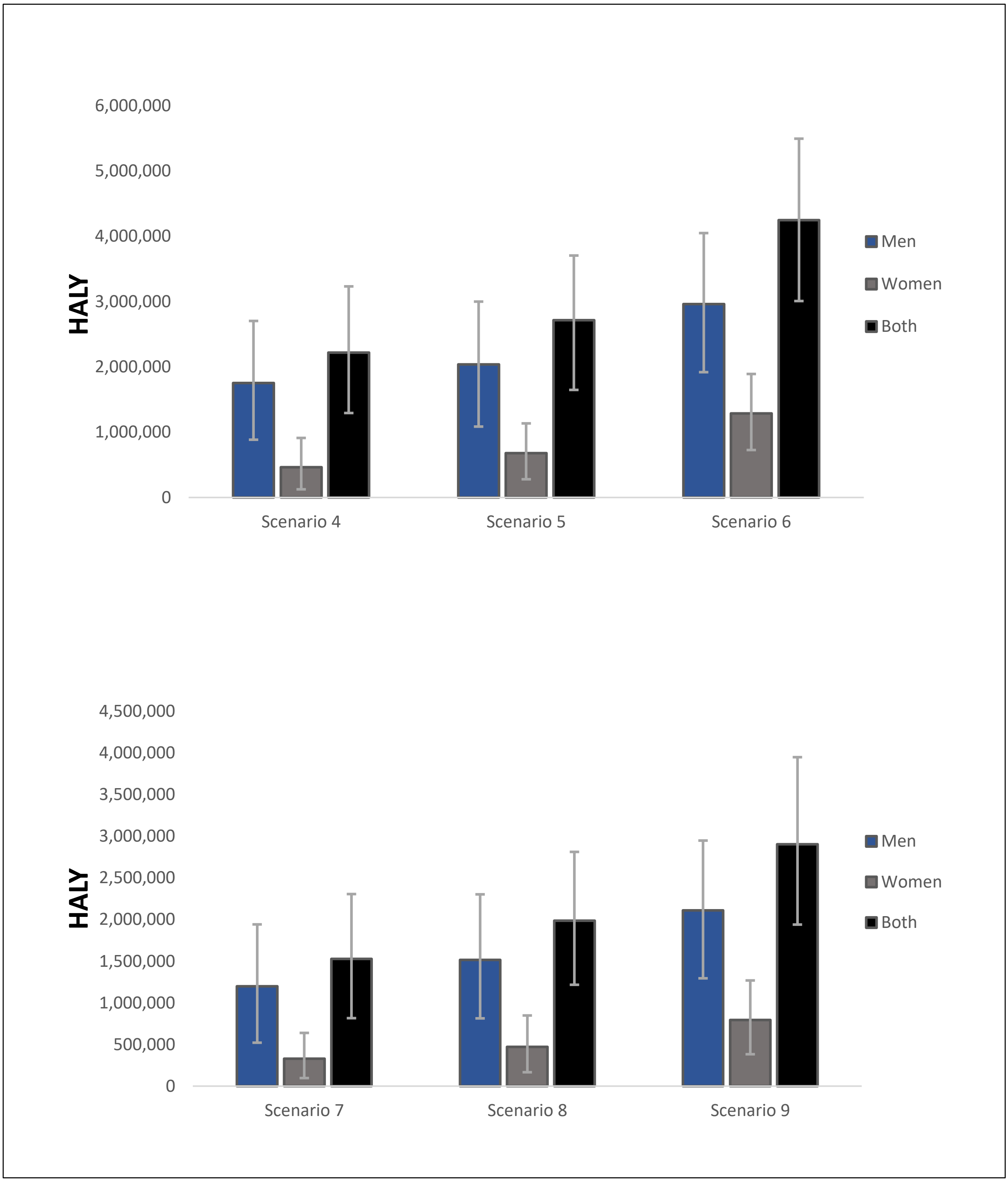

Figure 4: Health-adjusted life years (HALY) gained over the remaining lifetime under different scenarios

References

1. Ministry of Health, General Department of Preventive Medicine. National Survey on the risk factors of Non-communicable diseases (STEPS) Vietnam 2015.
2. He FJ, Li J, Macgregor GA. Effect of longer-term modest salt reduction on blood pressure. The Cochrane Database Sys Rev. 2013(4): Cd004937.
3. Pham TL. Occurrence, presentation, costs and three-month outcomes of Stroke in Viet Nam. PhD Thesis, Menzies Institute for Medical Research, University of Tasmania, Australia, 2015.
4. Global Burden of Disease Collaborative Network. Global Burden of Disease Study 2019 (GBD 2019) Results. Seattle, United States: Institute for Health Metrics and Evaluation (IHME), 2020. Available from http://ghdx.healthdata.org/gbd-results-tool.
